# The hepatoprotective effects of sitagliptin against cyclophosphamide-induced hepatotoxicity in rat

**DOI:** 10.22099/mbrc.2024.49925.1964

**Published:** 2024

**Authors:** Reza Maleki, Mohammad Foad Noorbakhsh, Nasrin Kazemipour, Maliheh Masoudian, Fatemeh Namazi, Saeed Nazifi

**Affiliations:** 1 Department of Basic Sciences, School of Veterinary Medicine, Shiraz University, Shiraz, Iran; 2 Molecular Department of Central Laboratory, School of Veterinary Medicine, Shiraz University, Shiraz, Iran; 3 Department of Pathobiology, School of Veterinary Medicine, Shiraz University, Shiraz, Iran; 4 Department of Clinical Sciences, School of Veterinary Medicine, Shiraz University, Shiraz, Iran

**Keywords:** Cyclophosphamide, Hepatotoxicity, Oxidative Stress, Sitagliptin

## Abstract

Hepatotoxicity is a serious side effects of cyclophosphamide. Thus, the present research investigates the protective properties of sitagliptin against cyclophosphamide-induced hepatotoxicity. Fifty male rats were randomly divided into five groups. They were pre-treated with either sitagliptin or normal saline once a day for the first ten days of the study. To induce acute hepatotoxicity, cyclophosphamide (200 mg/kg, i.p) was injected only one time and 45 min after the last dose of sitagliptin. The rats were sacrificed on the 11th day, and their blood and liver were collected for biochemical, gene expression, and histopathological assessments. Our results showed that cyclophosphamide induced obvious liver toxicity as marked by an increase in serum levels of alanine transaminase and aspartate transaminase, reduced serum albumin and total protein levels, in addition to histopathological changes. The malondialdehyde, tumor necrosis factor-α, and interleukin-6 levels were also elevated and total antioxidant capacity declined in serum and hepatic homogenates. Sitagliptin magnificently attenuated the cylophosphamide-induced histological alterations, improved liver function tests, enhanced total antioxidant capacity, and decreased malondialdehyde, tumor necrosis factor-α, and interleukin-6 in the blood and hepatic tissues. These findings suggest that sitagliptin has hepatoprotective activity against cyclophosphamide toxicity, which may be due to its antioxidant and anti-inflammatory effects.

## Introduction

Cyclophosphamide, or cytophosphane, is a synthetic oxazaphosphorine alkylating cytostatic agent, is approved by FDA in 1959 and widely used in the pharmacotherapy of various types of neoplasms, including solid tumors and hematological malignancies. It is also used as an immunosuppressant drug to treat some autoimmune diseases such as rheumatoid arthritis, multiple sclerosis, and systemic lupus erythematosus, as well as in organ transplantation [1, 2]. Despite the widespread benefits of cyclophosphamide in the remedy of malignancies and immune-mediated diseases, its use has been associated with several harmful fatal effects, one of the most important of which is hepatotoxicity, which limited its clinical use at the desired dosage and duration [3]. 

Several mechanisms have been proposed for cyclophosphamide-induced hepatotoxicity, among which oxidative stress has a more important and prominent role [4]. Cyclophosphamide is metabolized by the liver microsomal cytochrome P450 and converted to the two most active metabolites, phosphoramide mustard and acrolein, which triggers the genesis of free radicals and results in to oxidative stress, lipid peroxidation, alkylation of proteins and DNA, eventually resulting in cell apoptosis, tissue damage, fibrosis, cirrhosis, and organ failure [5]. Therefore, due to the negative effects of cyclophosphamide and its active metabolites (e.g., acrolein and free radicals) on cells, the search for a new compound with the ability to protect healthy cells and tissues against cyclophosphamide -induced toxicity is very important. In addition, it has been evidenced that antioxidant agents including vitamin E, gallic acid, berberine, punicalagin, and galangin can reduce cyclophosphamide-induced hepatotoxicity [6-10].

Sitagliptin is an anti-diabetic drug used to manage type 2 diabetes mellitus. It works by inhibiting the enzyme dipeptidyl peptidase-IV (DPP-4). By doing so, sitagliptin extends the post-prandial activity of two incretin hormones in the blood - glucagon-like peptide 1 (GLP1) and glucose-dependent insulinotropic polypeptide (GIP). This leads to a decrease in glucagon secretion and an increase in insulin secretion [11]. The hypoglycemic activity of sitagliptin may be due to the enhancement of bioavailability and function of GLP1 and GIP hormones [12]. Recently, the antioxidant and anti-inflammatory activity of Sitg has been reported in several studies [13-15]. Notably, sitagliptin has been evidenced to be hepatoprotective against methotrexate and aflatoxin B1-induced liver toxicity through inhibition of inflammatory and apoptotic processes [11, 16]. Therefore, due to the role of free radicals and inflammation in cyclophosphamide-induced hepatotoxicity and the antioxidant and anti-inflammatory effects of sitagliptin, the present study tried to explicate the probable hepatoprotective activity of sitagliptin against cyclophosphamide-induced liver toxicity.

## MATERIALS AND METHODS

**Drugs and kits: **Cyclophosphamide and sitagliptin were purchased from Sigma Chemical Co., located in Germany. The fasting blood sugar (FBS), aspartate aminotransferase (AST), Alanine Aminotransferase alkaline phosphatase (ALP), albumin (Alb), and total protein (TP) kits were purchased from Pars Azmoun, located in Tehran, Iran. Rat tumor necrosis factor-α (NF-α) and interleukin-6 (IL-6) ELISA Kits were obtained from Abcam Company, based in Cambridge, USA. The total antioxidant capacity (TAC) and malondialdehyde (MDA) kits were obtained from ZellBio GmbH Company in Ulm, Germany.

**Experimental Animals: **A total of 50 male Wistar rats, weighing 200 to 250 g, were purchased from the Pasteur Institute of Iran, Tehran, Iran. The animals were fed with standard mice food pellets and tap water *ad libitum* and acclimatized in a well-ventilated room with a 12 hours light/12 hours dark cycle and a normal temperature of 25±3°C. The animal handling procedures were conducted in conformity with the international guidelines for the care and use of experimental animals and approved by the local Research Ethical Committee at the Shiraz University, Iran. The study was conducted in accordance with the Basic & Clinical Pharmacology & Toxicology policy for experimental and clinical studies [17].

**Experimental procedures: **The dosage of cyclophosphamide used in this study was chosen based on previous research [1, 18]. In order to induce acute liver toxicity, a dose of 200 mg/kg of cyclophosphamide was injected intraperitoneally (i.p) 45 minutes after the last dose of either normal saline or sitagliptin on the tenth day of the study. Normal saline and sitagliptin were given orally (p.o.) once a day for the first ten days of the study. The animals were divided into five groups of equal size (n=10) as follows:

1. Control group: the animals were administered normal saline at a dose of 1 ml/kg orally
2. Cyclophosphamide group: the animals were administered normal saline (1 ml/kg, p.o.), and cyclophosphamide (200 mg/kg, i.p.)
3. Sitagliptin 30: the animals were administered sitagliptin (30 ml/kg, p.o), and cyclophosphamide (200 mg/kg, i.p)
4. Sitagliptin 60: the animals were administered sitagliptin (60 ml/kg, p.o), and cyclophosphamide (200 mg/kg, i.p)
5. Sitagliptin 120: the animals were administered sitagliptin (120 ml/kg, p.o), and cyclophosphamide (200 mg/kg, i.p)

**Sample Collection**: On the eleventh day of the study, the rats were given anesthesia using xylazine (10 mg/kg)/ketamine (80 mg/kg) (alfasan, Holland, i.p). Blood samples were then collected by cardiac puncture and centrifuged at 3000 rpm for 20 minutes at +4°C. The separated sera were stored at −80°C for future biochemical analysis. After sacrificing the animals, their livers were removed and divided into two parts. The first part was homogenized and kept in liquid nitrogen for further biochemical evaluations. The second part was fixed in 10% formalin for histopathological evaluations.

**Biochemical Assays: **The levels of FBS, ALB, TP, AST, ALT, and ALP in the serum were measured using standard kits and an autoanalyzer device. The TAC and MDA contents of both serum and liver homogenate were determined using commercial kits and the colorimetric method, following the manufacturer's instructions. Additionally, the levels of TNF-α and IL-6 in both serum and tissue were measured using ELISA kits, following the instructions provided with the kits.

**Histological assessments: **The first step in the process involved fixing the liver tissue samples in a 10% formalin solution. Once the samples were dehydrated using alcohol, they were then placed in paraffin blocks. After that, sections with a thickness of 5 microns were prepared using microtomes and stained with hematoxylin and eosin.

**Isolation of total RNA and synthesis of cDNA**: The animals were euthanized using CO2. Total RNA was extracted from 50 mg of liver tissue using Lysis buffer (Sinaclon, Iran) following the manufacturer's procedure. Potential DNA contamination was eliminated by treating the RNA (1 μg) with DNase I (1 U/μL) at 37°C for 30 minutes (Sinaclon, Iran). The purity and quantity of the extracted RNA were determined using a NanoDrop spectrophotometer at wavelengths of 120, 260, and 280 nm. To assess RNA purity, the optical density (OD) ratio at 260/280 nm was calculated, and samples with an OD ratio >1.8 were selected for reverse transcription. cDNA synthesis was performed using the Sinaclon first strand cDNA synthesis kit with random hexamer primers and 1 μg of RNA, following the kit's instructions (Sinaclon, Iran). During cDNA synthesis, a No-RT control was included in each run to check for genomic DNA contamination in real-time PCR. The cDNAs were stored at -20°C until they were used for real-time PCR.

**Real-time RT PCR analysis**: The relative quantifications of target genes (*TNFα *and* IL-6*) using real-time RT PCR test was conducted in a LightCycler 96 instrument (Roche, Germany). *GAPDH* gene was used as the reference gene. The specific sets of primers used for this experiment are presented in Table 1. 

The volume of the final master mix for gene expression analysis was 20 μl. It consisted of 4 μl of qPCR TM Green Master kit for EVA Green I (Solis BioDyne, Estonia), 2 μl of cDNA (~100 ng), 0.4 μl each of forward and reverse primers (200 nM), and 13.2 μl of nuclease-free distilled water. The PCR cycling conditions included an initial step at 95°C for 15 min, followed by 45 cycles at 95°C for 15 s and 60°C for 30 s. The real-time RT PCR reactions were performed in triplicate. Each run included a no template control (NTC) and a No-RT control. Additionally, a melting curve analysis was conducted to ensure the specificity of each PCR product. The efficiencies of the real-time RT PCR reactions were determined by analyzing a standard curve, which helped confirm that the amplification efficiencies of the primers used for the reference and target genes were similar. Finally, the 2^-ΔΔCt^ method was used to determine the gene expression ratios of the experimental group in comparison to the normal liver tissue.

**Table 1:** The primer sequence and amplicon size used in real-time PCR for the gene expressions

**Gene**			**Sequence**	**Amplicon size (bp)**	**Reference**
***TNFα***		Forward	5´GAATTACCTCCGAGATGACACC3´	148	Designed in
	Reverse	5´AGGCTGGTAGGAGTCTTTC3´	this study	
***IL-6***		Forward	5´TAGTCCTTCCTACCCCAACTTCC3´	76	Deng *et al*.
	Reverse	5´TTGGTCCTTAGCCACTCCTTC3´	(2017)	
***GAPDH***		Forward	5´ACTCACCTTTGGTCAATCCC-3´	146	Designed in
	Reverse	5´TCTTTAGGGATCCAGGCATTG3´	this study	

PCR: Polymerase Chain Reaction

**Statistical analysis: **The obtained values (Mean ± SEM) were compared using one-way analysis of variance (ANOVA), followed by the Tukey post hoc test, using SPSS 16.0 software. A significance level of p<0.05 was used to determine statistically significant differences for all comparisons.

## Results

As indicated in Table 2, injection of cyclophosphamide (200 mg/kg i.p.) on the tenth day of the study significantly increased the serum concentration of AST, ALT, and ALP compared to the control group, indicates that cyclophosphamide at the mentioned dose induced acute liver injury in these animals. Oral pre-treatment of rats with 30 mg/kg of sitagliptin considerably prevented cyclophosphamide-induced increase in AST, ALT, and ALP activity compared to the cyclophosphamide group. Additionally, the mean activity of these enzymes in group V (sitagliptin 120 mg/kg) was upper than group III (sitagliptin 30 mg/kg) and IV (sitagliptin 60 mg/kg). Also, intraperitoneal administration of cyclophosphamide significantly reduced the serum levels of total protein and albumin, which treatment with sitagliptin at doses of 30 mg/kg meaningfully prohibited their reduction. The serum glucose concentration was not significantly altered in comparison to other experimental groups.

**Table 2:** The effect of sitagliptin on serum biochemical parameters in cyclophosphamide-induced hepatotoxic rats

**Groups**	**AST (IU/L)**	**ALT (IU/L)**	**ALP (IU/L)**	**TP (mg/dl)**	**Alb (mg/dl)**	**FBS(mg/dl)**
**Control **	18.2±4.42	37.4±3.08	265.8±17.3	6.8±0.09	3.91±0.03	93.4±4.6
**Cyclophosphamide (** **NS + CP)**	170.2±22.89^a^	63.70±4.22^a^	397.2±28.39^a^	5.96±0.23^a^	3.55±0.09^a^	97.2±4.9
**Group III (Sitg 30 mg/kg + CP)**	116.4±3.14^b^	38.4±3.38^b^	273.7±15.2^b^	6.1±0.08^b^	3.72±0.05	96.1±6.4
**Group IV (Sitg 60 mg/kg + CP)**	132.8±3.58	43.3±3.12	300.5±30.44	5.96±0.18	3.6±0.12	94.9±7.33
**Group V (Sitg 120 mg/kg + CP)**	146.2±12.08	49.30±8.74	364.3±29.32	5.71±0.20	3.56±0.07^a^	92.01±6.5

Values are Mean ± S.E.M (n=10) Abbreviations: Alb; Albumin, ALP; Alkaline phosphatase, ALT; Alanine Aminotransferase, AST; Aspartate aminotransferase, CP; Cyclophosphamide, FBS; Fasting blood sugar, NS; Normal saline, Sitg; Sitagliptin, TP; Total protein. Analyzed by one-way ANOVA, followed by post hoc Tukey tests. ^a^p<0.05 vs. the control group, ^b^p<0.05 vs. the CP group.

Table 3 shows a notable increase in oxidative stress among rats treated with cyclophosphamide. Cyclophosphamide increased MDA levels and decreased TAC in the serum and liver tissue. Sitagliptin pre-treatment in cyclophosphamide-treated rats at doses of 30 /kg reduced MDA levels and enhanced the total antioxidant capacity in liver homogenates. Sitagliptin at the dose of 120 mg/kg decreased TAC contents in serum of cyclophosphamide-treated rats.

As presented in Table 4, cyclophsphamide caused an increase in TNF-α and IL-6 concentration in serum and hepatic tissue of rats. Pre-treatment of animals with sitagliptin (30 mg/kg) succeeded to counteract this increase and restoring their normal levels.

**Table 3:** The effect of sitagliptin on MDA and TAC in cyclophosphamide-treated rats

**Groups**	**Serum TAC** **(mmol/ml)**	**Liver TAC** **(mmol/g Tissue)**	**Serum MDA** **(µmol/ml)**	**Liver MDA** **(µmol/g Tissue)**
**Control**	0.47±0.02	0.79±0.04	53.94±4.21	142.2±5.17
**Cyclophosphamide (NS + CP)**	0.35±0.02^a^	0.61±0.03^b^	67.6±74.50	153.3±3.4
**Group III (Sitg 30 mg/kg + CP)**	0.39±0.02	0.86±0.03^c^	37.8±87.60^e^	108.8±6.14^bef^
**Group IV (Sitg 60 mg/kg + CP)**	0.39±0.02	0.83±0.03^d^	41.69±10.56	137.8±4.5
**Group V (Sitg 120 mg/kg + CP)**	0.33±0.01^b^	0.75±0.05	42.87±2.00	146.0±10.52

Values are Mean ± S.E.M (n = 10) Abbreviations: MDA; malondialdehyde, TAC; Total antioxidant capacity, CP; Cyclophosphamide, Sitg; Sitagliptin. Analyzed by one-way ANOVA, followed by post hoc Tukey tests. ^a^p<0.01 vs. control group, ^b^ p<0.05 vs. the control group, ^c^p<0.001 vs. the CP group, ^d^ p<0.01 vs. the CP group, ^e^ p<0.05 vs. the CP Group, ^f^p<0.01 vs. group V

**Table 4:** The effect of sitagliptin on levels of TNF-α and IL-6 in cyclophosphamide-induced hepatotoxic rats

**Groups**	**Serum TNF-α** **(pg/ml)**	**Tissue TNF-α** **(pg g tissue)**	**Serum IL-6** **(pg/ml)**	**Tissue IL-6** **(pg/g tissue)**
**Control**	285.2±17.35	244.81±6.14	12.98±0.59	8.62±0.36
**Cyclophosphamide (** **NS + CP)**	367.18±18.82^a^	320.4±21.76^a^	17.91±2.11^a^	10.72±0.39^a^
**Group III (** **Sitg 30 mg/kg + CP)**	264.9±19.06^b^	213.6±9.02^b^	11.31±0.33^b^	8.64±0.71^bc^
**Group IV (** **Sitg 60 mg/kg + CP)**	308.6±8.11	243.7±5.16^c^	13.16±0.43	9.64±0.49
**Group V (** **Sitg 120 mg/kg + CP)**	337.7±22.63	256.8±19.19	13.73±0.36	10.82±0.36^a^

**Figure 1:**
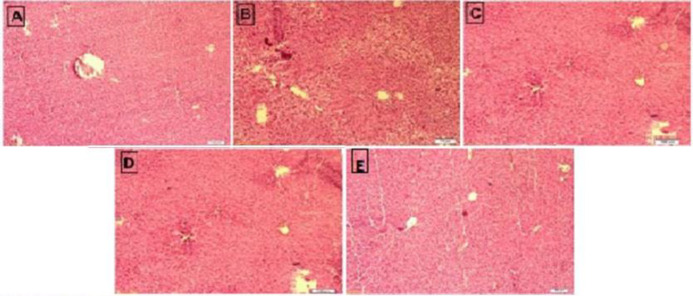
Effect of sitagliptin on cyclophosphamide-induced liver toxicity in rats. Intoxication with cyclophosphamide made histopathological changes such as dilatation and congestion in the central vein, infiltration of leucocytes, sinusoidal dilatation, and vacuolation in hepatocytes (B). Pre-treatment of rats with sitagliptin at doses of 60 and 120 mg/kg reversed the cyclophosphamide-induced liver damage towards normal (D, E). A: Control group, B: cyclophosphamide group, C: cyclophosphamide + sitagliptin 30 mg/kg, D: cyclophosphamide + sitagliptin 60 mg/kg, and E: cyclophosphamide + sitagliptin 120 mg/kg (40X).

Values are Mean ± S.E.M (n = 10) Abbreviations: IL-6; Interleukin-6, TNF-α; Tumor necrosis factor-alpha, CP; 200 mg/kg of Cyclophosphamide, Sitg; Sitagliptin. Analyzed by one-way ANOVA, followed by post hoc Tukey tests. ^a^ p<0.005 vs. the control group, ^b^ p<0.01 vs. the CP group, ^c^ P<0.05 vs. group V,

Histopathological examination of the hepatic tissue indicated that in the control group, there is a normal histoarchitecture of liver and cellular structures (Fig. 1A). The liver photomicro-graphs of the ctclophosphamide-treated group showed alteration in liver architecture such as dilatation and congestion in the central vein, periportal leucocyte infiltration, and sinusoidal distention. Enlarged nuclei and vacuolation also were observed in hepatocytes (Fig. 1B). However, orally pre-treatment of rats with sitagliptin mitigated these pathological alterations. The hepatic photomicrographs of stig pre-treated rats (60 and 120 mg/kg) exhibited a similar histoarchitecture and cellular structure to the control group (Fig. 1D, E). 

**Figure 2:**
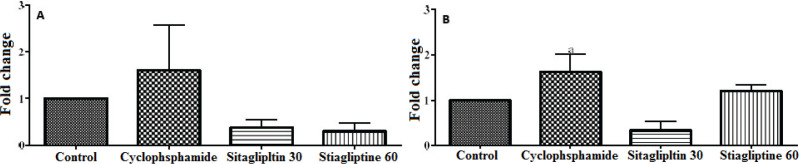
Gene expression ratio of TNF-α (A) and IL-6 (B) in the liver tissue of the different groups. Data are expressed as means±SEM. a: significant against sitagliptin 30, p< 0.05

Based on real-time PCR results, we observed that the expression of *TNF-α* gene decreased in rats treated with sitagliptin 30 mg/kg compared to rats that received cyclophosphamide, and this reduction was increased by the administration of 60 mg/kg of sitagliptin. Due to the administration of cyclophosphamide, the expression of *IL-6* gene increased, and this gene was decreased by the administration of 30 mg/kg of sitagliptin. By increasing the dose of sitagliptin to 60 mg/kg, the expression of *IL-6* gene increased (Fig. 2).

## DISCUSSION

Cyclophosphamide is an alkylating agent commonly used to treat autoimmune diseases and various cancers. Previous studies have shown that in addition to therapeutic effects, cyclophosphamide has to severe toxic effects on healthy organs, especially the liver, which has limited its use [3]. 

In the present study, treatment of rats with cyclophosphamide increased serum levels of hepatic enzymes such as AST and ALT and decreased serum concentrations of total protein and albumin, which confirms acute liver injury. In addition, the results of histopathological studies also confirmed liver damage. These results are consistent with previous studies. Doustimotlagh et al. demonstrated that intoxicating rats with cyclophosphamide raises serum levels of AST and ALT while decreasing Alb and TP levels [1]. Elevated serum ALT and AST levels in cyclophosphamide-induced hepatotoxicity are due to cell damage and loss of hepatocyte membrane integrity as these cytosolic enzymes are released into the bloodstream [9]. Decreasing the serum level of liver enzymes and preventing histological changes with sitagliptin shows that this drug can prevent cyclophosphamide-induced liver damage by stabilizing the cell membrane of hepatocytes.

Mustard phosphoramide and acrolein are two active metabolites of cyclophosphamide which triggers the production of highly reactive oxygen species, membrane lipids peroxidation, production of MDA, and decreased natural antioxidant capacity in blood and tissues [18]. Fouad (2016) reported that cyclophosphamide injection induces a notable elevation in hepatic MDA and a remarkable reduction in liver TAC [8]. Hamzeh et al. (2018) also found an augmented level of MDA after cyclophosphamide administration [19]. In line with these results, our finding indicated that injection of cyclophosphamide produced a noticeable oxidative stress as marked by considerable increased MDA levels and reduced TAC in serum and liver tissues, whereas sitagliptin significantly inhibited cyclophosphamide-induced changes in MDA and TAC, which could be explained by the antioxidant and free radical scavenging activity of sitagliptin, as demonstrated in several previous studies. Abo-Hadad et al. (2017) demonstrated that orally pre-treatment of sitagliptin diminished MDA content and improved the antioxidant capacity of the liver in methotrexate-treated rats [11]. 

To gain further insight into the hepatoprotective properties of sitagliptin, we examined its effect on serum and liver levels of the two pro-inflammatory cytokines TNF-α and IL-6. Our finding exhibited that cyclophosphamide significantly increases the serum and hepatic concentration of TNF-α and IL-6. As a result of cyclophosphamide administration, gene expression of these inflammatory factors increased. As support, recent evidence indicates that inflammation is a key factor in the development of hepatotoxicity caused by cyclophosphamide [20, 21]. The release of these cytokines was reported to contribute to the severity of liver injury [22]. Our finding indicated that pre-treatment of the rats with sitagliptin notably decreased the TNF-α and IL-6 concentration in serum and liver tissues. And also the gene expression of these inflammatory factors decreased. These findings are consistent with earlier studies. Hewedy (2021) showed that the pretreatment with sitagliptin for five days significantly decreased serum levels of TNF-α and IL-1β in acetaminophen-treated mice [22]. In another research, it has been shown that sitagliptin counteracts with TNF-α, IL-6, and IL-1β increase in methotrexate-treated rats [11]. 

Our findings revealed that prophylactic administration of sitagliptin can combat cyclophosphamide-induced liver toxicity through the enhancement of antioxidant capacity, inhibition of the lipid peroxidation, and pro-inflammatory cytokines. Further researches are needed to explain the exact mechanism of the protective effects of sitagliptin against cyclophosphamide hepatotoxicity.
